# Postnatal Care Service Utilisation in Ethiopia: Reflecting on 20 Years of Demographic and Health Survey Data

**DOI:** 10.3390/ijerph18010193

**Published:** 2020-12-29

**Authors:** Tensae Mekonnen, Tinashe Dune, Janette Perz, Felix Akpojene Ogbo

**Affiliations:** 1 Translational Health Research Institute (THRI), School of Medicine, Western Sydney University, Penrith NSW 2751, Australia; t.dune@westernsydney.edu.au (T.D.); j.perz@westernsydney.edu.au (J.P.); f.ogbo@westernsydney.edu.au (F.A.O.); 2 School of Science and Health, Western Sydney University, Penrith, NSW 2751, Australia

**Keywords:** postnasal care, urban–rural variation, Ethiopia, Demographic and Health Survey

## Abstract

Background: Most maternal deaths in the world occur during the postpartum period, especially within the first two days following delivery. This makes postnatal care (PNC) critical to improving the chances of maternal and child survival. Over the past 20 years, the proportion of women receiving antenatal care (ANC) in Ethiopia has increased while the proportion of those receiving PNC has remained low. This study aimed to understand the trends, determinants and urban–rural variations of PNC service utilisation. Methods: This study draws on the Ethiopian Demographic and Health Survey (EDHS) data for the years 2000 (n = 4552), 2005 (n = 4467), 2011 (n = 4445) and 2016 (n = 4275) to estimate the trends and determinants of PNC service utilisation. Multivariate logistic regression models with adjustment for clustering and sampling weights were used to investigate the association between the independent factors, the study factors and PNC service utilisation. Results: Over the twenty-year period of the EDHS, the proportion of Ethiopian women who received PNC services increased from 5.6% (95% CI: 4.6–6.9%) in 2000 to 18.5% (95% CI: 16.4–20.7%) in 2016. Similarly, women who received PNC services in urban areas increased from 15.2% (95% CI: 23.6–30.7%) in 2000 to 47% (95% CI: 60.4–67.3%) in 2016. Women who were in the wealthy quintile, had ANC visits, delivered in a health facility, and delivered by caesarean section were most likely to have PNC. The present study also showed that whilst birth spacing was a significant factor among urban women, wealth index, ANC visits, and perception of health facility distance were significant factors among rural women. Conclusions: The study suggests low levels of utilisation of PNC among Ethiopian women from rural districts. Geographically targeted interventions with a focus on low-socioeconomic rural women, and those with no previous contacts with the health system during pregnancy, are needed to improve PNC in Ethiopia.

## 1. Introduction

Ethiopia is the second most populous country in Africa and has one of the highest rates of maternal mortality (401 deaths/100,000 live births in 2017) in the world [1,2,3]. Past research shows that most maternal deaths occur in the postpartum period as a result of haemorrhage, hypertensive disorders of pregnancy, abortion and sepsis [1]. Maternal mortality is a preventable global tragedy and proven and cost-effective interventions such as antenatal care (ANC), skilled delivery and postnatal care (PNC) have been reported to reduce morbidity and mortalities related to pregnancy and childbirth [4]. For instance, Bhutta and colleagues suggest that the provision of quality and effective care for women delivering in facilities could prevent an estimated 113,000 maternal deaths [5].

According to World Health Organization (WHO), a majority of maternal deaths in the world occur during the postpartum period, especially within the first two days after delivery, that makes the postnatal period critical to improving maternal and child survival [6]. The postpartum period is the period beginning 1 h after the delivery of the placenta and continuing until 6 weeks (42 days) after delivery [7]. Early PNC provides a window of opportunity for the identification and management of complications that may occur at the time of labour, delivery or immediately after delivery [7]. Health care providers will also counsel the mother about maternal nutrition, breastfeeding, immunisation and other childcare services. Furthermore, stress relieving psychological and emotional supports can also be provided [7,8].

Over the past 25 years, several programs have been developed and implemented to improve maternal health service utilisation in Ethiopia. Even though the proportion of women receiving ANC in Ethiopia is high [9], the proportion of women delivering by the assistance of a skilled attendant and having PNC remained low [10,11]. Research indicates that factors across individual through to health system levels contribute to this disparity between ANC and PNC. For instance, the 2011 Ethiopian Demographic and Health Survey (EDHS) revealed that fewer mothers living in rural areas received PNC compared to those living in urban areas (3% vs. 32%) [12]. According to Koblinsky and colleagues [13], the traditional home confinement of women after childbirth in Ethiopia contributes to the low use of PNC services. Others also reported significant regional variations in the use of ANC in Ethiopia with low levels reported in Somali, Oromia, Gambella and Southern Nations, Nationalities, and People’s Region (SNNPR) [14]. These findings imply the need for national and regional level interventions that target individual women, their families and surrounding communities as well as health care professionals and the wider health care system to improve the utilisation of maternity care services in Ethiopia.

There have been some studies that examined why PNC service utilisation remains low in Ethiopia. Some of these studies had a small sample size [15,16] and others covered a very small geographical area [17,18] that may put the representativeness of the studies and generalisability of the findings into question. Although there have been four rounds of the EDHS, studies that used these data have concentrated on the 2016 survey [19,20]. This makes investigating the determinants of PNC use in Ethiopia using the four nationally representative and relatively large sample surveys critical to identifying possible interventions. Results of this analysis can inform interventions that aim to improve the implementation and utilisation of maternal health services in Ethiopia and achieve the sustainable development goal 3 (SDG goal 3) of reducing the global maternal mortality ratio to less than 70 per 100,000 live births by 2030 [21]. According to WHO, this goal can be achieved by improving access to quality reproductive and maternal health services, ensuring universal health coverage for comprehensive reproductive and maternal health care, and addressing all causes of maternal morbidity and mortality [4]. Therefore, this study aimed to (1) examine the trends of PNC utilisation; (2) identify factors associated with the use of PNC, and (3) compare urban–rural variations of PNC use.

## 2. Materials and Methods

### 2.1. Data

The 2000–2016 EDHS data were used. EDHS is designed to estimate key national level population, health and nutrition indicators for program planning, monitoring and evaluation. Main advantages of EDHS data include high sample size and response rate, coverage of all regions and city administrations, and high standard data collection tools [22]. In the present study, a total of 17,740 (4552 in 2000, 4467 in 2005, 4445 in 2011 and 4275 in 2016) responses were analysed. All analysis was weighted to ensure actual representativeness of the data and to allow us draw nationally generalisable conclusions. Weighting was needed because some areas were oversampled to produce estimates for all the nine regions and two city administrations.

### 2.2. Study Setting

The Federal Democratic Republic of Ethiopia has nine regional states, two city administrations, 611 weredas (districts) and 15,000 kebeles. Regions are divided into zones, and zones, into administrative units called weredas. Each wereda is further subdivided into the lowest administrative unit called kebele (Population Census Commission, 2008). Ethiopia is the second most populous nation in Africa, with nearly 115 million people based on the 2019 United Nations estimate, after Nigeria with over 180 million people [23]. Males represent 50.5% of the population and females 49.5, with 21% being of reproductive age (15–49 years). Although health service coverage reaches 92% of the population, the utilisation of maternal healthcare services is low. The 2016 EDHS indicated that 62% of pregnant women received ANC, 26% of pregnant women delivered in a health facility, and 17% received PNC [24]. About 25% and 35% of all reproductive age women and married women use contraception, respectively.

### 2.3. Outcome Variable

The utilisation of postnatal care is the outcome variable in this study. PNC attendance (utilisation) was defined in this study as having at least one visit within the first 42 days (six weeks) of birth [25]. In the survey, this question was asked: After you gave birth, did anyone check on your health?” Response options included: “Yes” and “No”, to determine if the mother had attended any PNC visitor check-ups [24]. From the response, we used Yes = 1, and No = 0 for our analysis.

### 2.4. Independent Variables

**Figure 1:**
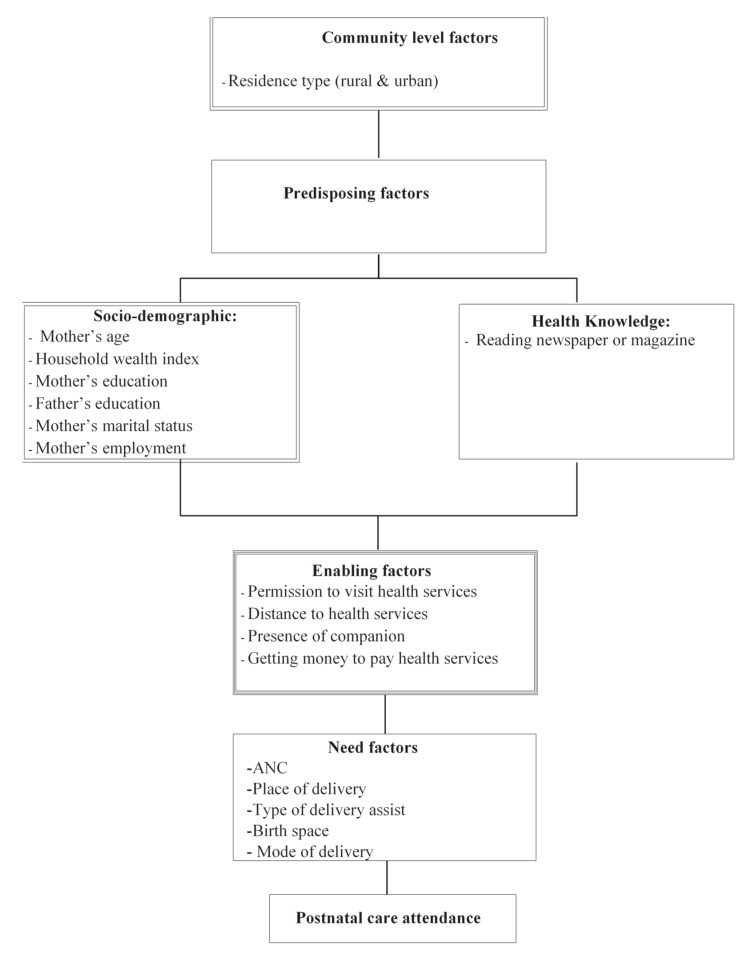
Conceptual framework.

Independent variables in this study were chosen based on previous research on the topic [26]. Anderson’s behavioural framework [27] (see Figure 1) was used to organise the determinants of PNC utilisation. The factors assumed to influence the use of PNC were categorised into four main factors: community level, predisposing, enabling and need factors. Community level factors include geopolitical zone and residence type. Predisposing (socio-demographic and health knowledge factors) include maternal age, household wealth index, maternal and paternal education, marital and employment statuses and reading magazine or newspaper. Need factors include contraceptive use and future plan to have a child. Variables such as permission to visit health services, distance from health services, presence of companion and getting money to pay for health services are categorised as enabling factors.

### 2.5. Statistical Analysis

The analysis started with the calculation of simple frequencies and percentages for study variables for the total population, urban and rural residence location groups. A series of frequencies and cross tabulations were conducted to estimate the prevalence of PNC service by the study factors for the three locations. Univariate logistic regression analysis was conducted to examine factors associated with PNC service in Ethiopia and rural–urban differences. Variables with *p* value < 0.05 in univariate models were entered into multivariate models. Crude and adjusted odds ratios (ORs) and their 95% confidence intervals were reported in the present study for location groups. All analyses were performed using the “svy” command for calculation of counts and percentages in Stata 15.0 (Stata Corporation, College Station, TX, USA) to adjust for sampling weight, clustering and stratification.

### 2.6. Ethics

This study used secondary data made publicly available by ICF International and the EDHS. The authorisation for using the data in the current study was granted from the DHS program upon presenting the aims of the study and the research plan. Detailed information on the data collection procedures employed by EDHS has been published as a full report elsewhere [12,24].

## 3. Results

### 3.1. Background Characteristics of the Study Population

The study included a total weighted sample of 17,740 reproductive age women representing the nine regions and two city administrations of Ethiopia. The majority (72%) had no formal education and low (60.2%) wealth status. Nearly half (48%) of the participants were between 25 and 34 years. During their last pregnancy 30.9% of the women had ANC visits and the majority (85%) gave birth at home. Over two-thirds of the women (69.9%) reported that distance from a health facility is a big problem to access services (Table 1).

### 3.2. Utilisation of PNC Services in Ethiopia

**Figure 2:**
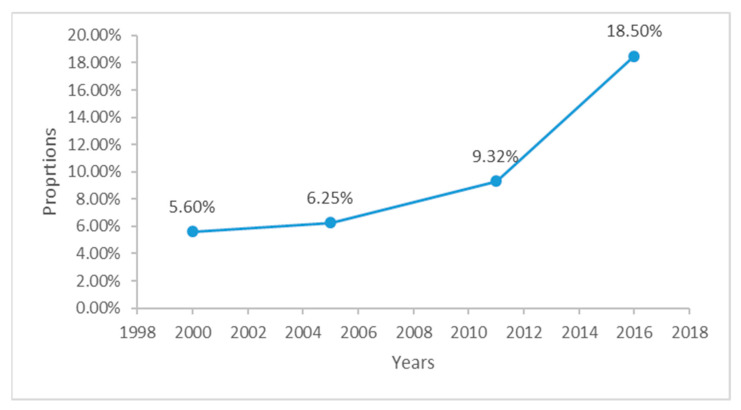
Trends of postnatal care (PNC) utilisation in Ethiopia (EDHS) 2000–2016.

The analysis revealed that the use of PNC services increased from 5.6% to 18.5% over the 20-year period of the EDHS (Figure 2).

**Figure 3:**
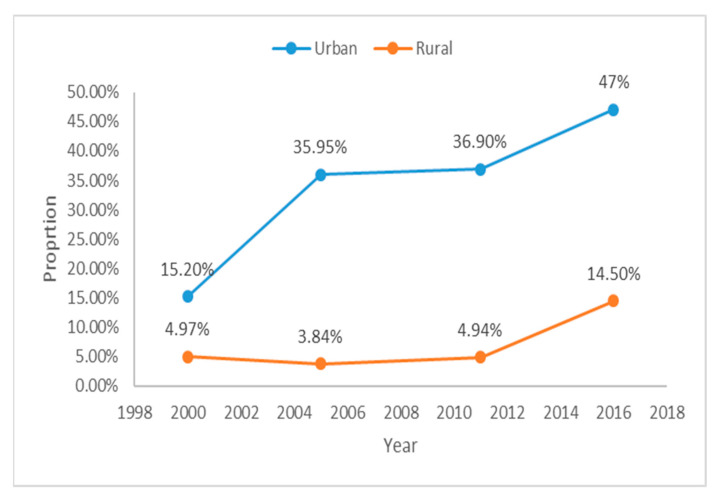
Trends of PNC in urban and rural women of Ethiopia (EDHS) 2000–2016.

A significant variation in the use of PNC services between urban and rural areas was also identified. Whilst PNC use increased from 15.2% to 47% in Urban areas (Figure 3), the increase in rural areas was observed mainly during the 2016 survey.

### 3.3. Determinants of PNC in Ethiopia

Multivariate analysis revealed several factors that influence the use of PNC in Ethiopia (Table 2). From 2000 to 2016, mothers who had ANC visits during pregnancy were more likely to utilise PNC services as well. This association is positive for those women who had 1–3 (OR = 1.9, 95% CI [1.36–2.66] Table 3) and 4+ ANC visits (OR = 3.01, 95% CI [2.15–4.21]). The likelihood of having PNC was higher among urban women (OR = 2.99, 95% CI [1.43–3.45]) and those who delivered their babies by a caesarean section (OR = 4.3, 95% CI [2.39–7.71]). Health facility delivery was the other factor that significantly influenced the chance of women receiving PNC care with those women who delivered their babies in health facilities were about 2.5 times more likely to have PNC care (OR = 2.5, 95% CI [1.04–6.25]) compared to women who delivered at home. Over the 20-year period, mothers in the high-wealth quintile had higher odds of receiving PNC care compared with those who were from low socioeconomic households (OR = 1.57; 95% CI [1.12, 2.20]). Furthermore, mothers whose husbands had professional or semi–professional jobs were highly likely to utilise PNC services after the delivery of their babies (OR = 1.52; 95% CI [1.00, 2.34]). Finally, women who perceived that “distance from a health facility is not a big problem” were significantly likely to have engaged with PNC services (OR = 1.29; 95% CI [1.02, 1.62]).

### 3.4. Determinants of PNC Service Utilisation in Urban–Rural Ethiopia

Comparative multivariate analysis of the data revealed critical urban rural differences in factors that influence the use of PNC services (Table 3). Among the rural population, higher wealth increased the odds of having PNC services (AOR: 1.85, 95% CI: 1.23–2.79; *p* < 0.001 for wealthy category). Both rural and urban women who delivered their babies by caesarean section were more likely to have engaged with PNC services (AOR: 7.4, 95% CI: 2.51–21.68, *p* = 0.00 for rural and AOR: 3.5, 95% CI: 1.93–6.68, *p* = 0.00 for urban). Rural mothers with husbands who had a professional or semi–professional job were significantly likely to utilise PNC compared to those who had no employment (AOR: 1.96, 95% CI: 1.13–3.39, *p* = 0.00). The association between birth spacing of greater than 24 months and PNC service utilisation was significant for urban mothers (AOR: 1.78, 95% CI: 1.08–2.96; *p* < 0.001). Compared to rural women who had no ANC visits during pregnancy, those who had received ANC were significantly likely to also have PNC as well (AOR 2.28, 95% CI: 1.58–3.33, *p* = 0.00 for 1–3 ANC visits, and AOR 2.28, 95% CI: 1.58–3.33, *p* = 0.00 for 4^+^ visits). Distance from a health facility was another factor that showed significant association with PNC service utilisation among rural women. Those who had a perception that “distance is not a big problem” were more likely to have PNC (AOR 1.35, 95% CI: 1.03–1.77, *p* = 0.02).

## 4. Discussion

This analysis revealed that the use of PNC care has increased over the 20-year period of the EDHS although the proportion remains low. Findings also identified a substantial urban–rural difference among Ethiopian women’s engagement with PNC care. In addition, this study identified that PNC service utilisation was significantly influenced by predisposing and need factors of Anderson’s model of health behaviour. Accordingly, the likelihood of women to receive PNC service was higher among those who were from the rich wealth quintile, had ANC visits, delivered in a health facility, and delivery by caesarean section. Furthermore, the present study also showed that whilst birth spacing was a significant factor among urban women wealth index, ANC visits, and perception of a health facility’s distance were significant among rural women. The identification of these factors is critical for improving PNC service utilisation in Ethiopia and other developing countries.

An interesting finding of this study is the effects of need factors such as ANC attendance and delivery in a health facility on PNC service utilisation. The analysis found that mothers who had 1–3 ANC visits, and four or more visits as recommended by the WHO [28], were more likely to have PNC visits. Consistent with findings from Nigeria, Nepal and Ethiopia [29], our analysis also revealed that women who delivered in a health facility and by a caesarean section were significantly likely to use PNC services as well. The finding suggests that women are more likely to receive PNC service if they had interventions that improve access to and utilisation of ANC and skilled delivery care—this finding is consistent with studies from around the world [30]. The finding that urban women with over 24 months of birth spacing were more likely to use PNC implies the importance of family planning and maternal health service integration [31].

In this study, we showed that women who perceived that distance to the health facility was not a big problem were significantly less likely to use PNC care, suggesting that distance to the health facility and the associated transportation cost could be a barrier for some women to access PNC. This finding was in line with those of other studies in Ethiopia [32] and elsewhere [33], and suggests that physical proximity to health facilities and geographical distance have a significant role in the utilisation of PNC services. Consistent with other studies [33] rural women in the higher wealth index were significantly likely to use PNC services than those who were from poor categories. In Ethiopia, although maternal health services are free of charge [34,35], the cost of travel and/or medicines is out-of-pocket, which has been showed to act as barriers to full access to maternal health services in the country. These findings imply interventions that aim to improve women’s engagement with PNC care should focus on low socioeconomic rural women living far from health facilities.

This study has some strengths and limitations that should be considered when interpreting the findings. This study involved a nationally representative and relatively larger sample size compared to other cross-sectional studies in Ethiopia which implies that generalisation of the findings could be possible. Most of the study findings also support the conclusions from smaller studies which again demonstrates the national representativeness of the data. However, the cross-sectional nature of the data means that temporal association between the study variables and PNC cannot be established. In addition, self-reporting was the method employed during EDHS data collection which suggests that recall bias is a possibility which may lead to under- or over-estimation of proportions and associations between exposure and outcome variables. Furthermore, as this study relied on secondary analysis of EDHS data we could not see if variables not measured by the EDHS such as partner support, cultural practices, health status of women, transportation, health professional attitude and skills would influence the women’s engagement with PNC care, and this warrants the need of additional primary studies. Qualitative studies examining the women’s experiences of receiving PNC may also shed light on how these factors operate to influence the women’s access to care after childbirth.

## 5. Conclusions 

Overall, the present study indicated the low level of utilisation of PNC among Ethiopian women with significant urban–rural variations. The study also demonstrated that ANC follow up, delivery by a cesarean section, perceived distance from a health facility and being from the high-wealth quintile are important determinants to seek PNC services in both urban–rural areas and at the national level in Ethiopia. It is important that efforts that aim to improve PNC utilisation should be area specific and focus on low socioeconomic rural women and those with no contact with the health system during pregnancy. A combination of both community and facility level interventions are also essential to improve maternal health service utilisation in Ethiopia.

## Figures and Tables

**Table 1:** Prevalence of postnatal care by study factors in Ethiopia (Ethiopian Demographic and Health Survey (EDHS)) 2000–2016.

Total Population				Urban			Rural		
Variables	N	Prevalence (95%CI)	*p* Value	N	Prevalence (95%CI)	*p* Value	N	Prevalence (95%CI)	*p* Value
PNC Yes	1744	9.82(9.03–10.6)	*p* < 0.001	636	9.8(9.03–10.6)	*p* < 0.001	1107	6.24(5.6–6.90)	*p* < 0.001
No	15,997	90.17(89.3–90.9)	*p* < 0.001	1132	90.17(89.3–90.9)	*p* < 0.001	14,864	90.17(89.3–90.9)	*p* < 0.00
**Predisposing factors**									
**Maternal education**									
No education	12,799	72 (72.5–73.6)	*p* < 0.001	575	32.5(28.5–36.8)	*p* < 0.001	12,223	76.5(75–78)	*p* < 0.001
Primary	4029	22.7(21.4–24.3)		609	34.4(31–37.9)		3421	21.4(20–23)	
Secondary	912	5.1(4.5–5.8)		584	33(29.5–36.6)		328	2(1.7–2.4)	
**Maternal working status**									
Not working	9753	57.8(55.9–59.6)	*p* < 0.001	902	51.2(46.8–55.5)	*p* = 0.142	8851	58.5(56.5–60.6)	*p* < 0.01
Professional	2146	12.7(11.6–13.8)		604	34.2(30–38.7)		1543	10(9.1–11.3)	
Agricultural	4968	29.4(27.6–31.3)		256	14.5(11.2–18.6)		4713	31(29.2–33.2)	
**Partner education**									
No education	9615	54.5(52.8–56.2)	*p* < 0.001	391	22.2(19.4–25.3)	*p* < 0.001	9224	58(56.3–59.8)	*p* < 0.001
Primary	6116	34.6(33.1–36.2)		559	31.8(28.4–35.5)		5557	35(33.3–36.6)	
Secondary	1906	10.8(9.9–11.7)		805	45.8(41.9–49.8)		1100	7(6.2–7.6)	
**Partner working status**									
Not working	567	3.25(2.7–3.8)	*p* < 0.001	91	5.3(3.7–7.5)	*p* = 0.052	477	3(2.4–3.6)	*p* < 0.001
Professional	1856	10.6(9.7–11.6)		841	49.6(45.9–53.3)		1015	6.4(5.8–7.1)	
Agricultural	14,990	86(84.9–87.1)		762	45(41–48.9)		14,227	90.5(89–91)	
**Household wealth index**									
Poor	10,289	60.2(58–62)	*p* < 0.001	110	6.5(4.6–9.19)	*p* < 0.001	10,179	66(63.9–68)	*p* < 0.001
Middle	4584	26.8(25.2–28.5)		224	13.4(10.3–17.1)		4360	28.2(26.5–30)	
Rich	2210	12.9(11.6–14.3)		1338	80(76–83.5)		871	5.6(4.8–6.5)	
**Mother’s age**									
15–24	5595	31.5(30.4–32.6)	*p* = 0.014	523	29.5(26–33.2)	*p* = 0.017	5072	31.7(30–32)	*p* = 0.189
25–34	8521	48(46.8–49.2)		978	55.3(51.9–58.6)		7543	47.2(46–48)	
35–49	3624	20.4(19.5–21.3)		267	15(13.2–17.2)		3356	21(20–22)	
**Marital status**									
Never married	109	0.61(0.46–0.81)	*p* = 0.003	33	1.86([1.2–2.8)	*p* = 0.209	76	0.47(0.33–0.67)	*p* = 0.040
Currently married	16,720	94.2(93.6–94.8)		1579	89.3(86.6–91.5)		15,140	94(94–95)	
Formerly married	911	5.13(4.6–5.7)		156	8.8(6.7–11.3)		755	4.7(4.2–5.3)	
**Reading magazine**									
Yes	358	7.79(7.12–8.51)	*p* < 0.001	261	14.9(12.4–17.8)	*p* < 0.001	94	0.59(0.44–0.79)	*p* < 0.001
No	1382	2.02(1.70–2.38)		371	21(17.8–24.4)		1011	6.33(5.71–7.01)	
**Need factors**									
**ANC visit**									
None	10,796	61(59.1–62.8)	*p* < 0.001	459	26.1(21.6–31.1)	*p* < 0.001	10,337	64.8(62.9–66.6)	*p* < 0.001
1–3	3901	22(20.9–23.2)		436	24.8(21.4–28.5)		3465	21.7(20.5–22.9)	
Four or more	3001	16.9(15.7–18.2)		862	49(44.1–53.9)		2140	13.4(12.2–14.6)	
**Place of delivery**									
Home	15,354	86.5(85–87.8)	*p* < 0.001	797	45(39.1–51)	*p* < 0.001	14,558	91(89.8–92.3)	*p* < 0.001
Health facility	2385	13.4(12.1–14.9)		971	54.9(48.9–60.8)		1414	8.8(7.7–10.1)	
**Type of delivery assist**									
Health professional	2468	13.9(12.5–15.3)	*p* < 0.001	1008	57(50.9–62.8)	*p* < 0.001	1461	9.1(7.9–10.4)	*p* < 0.001
Traditional birth attendant (TBA)	5493	30.9(29–32.8)		362	20.4(16.4–25.2)		5131	32(30.2–34)	
Other non-health professional	9779	55.1(53–57.1)		398	22.5(18.6–26.9)		9380	58(56–60)	
**Birth interval**									
No previous birth	3273	18.4(17.6–19.3)	*p* < 0.001	531	30.1(26.6–33.9)	*p* = 0.005	2741	17.1(16.3–18.03)	*p* < 0.001
<24months	2378	13.4(12.5–14.3)		181	10.2(8.4–12.3)		2197	13.7(12.8–14.7)	
≥24 months	12,074	68(67–69)		1050	59.5(55.5–63.5)		11,024	69.0(67.9–70.1)	
**Enabling factors**									
**Accompany for medical help**									
Big problem	7798	59.1(57.2–61)	*p* < 0.001	483	33.1(29.1–37.3)	*p* = 0.138	7315	62.3(60.3–64.3)	*p* < 0.001
Not a big problem	5388	40.8(38.9–42.7)		976	66.8(62.6–70.8)		4412	37.6(35.6–39.6)	
**Distance from the health facility**									
Big problem	9226	69.9(67.9–71.8)	*p* < 0.001	402	27.6(23.9–31.6)	*p* = 0.013	8823	75.2([73.3–77.06)	*p* < 0.001
Not a big problem	3959	30(28.1–32)		1054	72.3(68.4–76.03)		2905	24.7(22.9–26.6)	
**Permission for medical help**									
Big problem	4790	36.3(34.4–38.3)	*p* < 0.001	260	17.8(14.4–21.7)	*p* = 0.234	4530	38.6([36.5–40.7)	*p* = 0.003
Not a big problem	8394	63.6(61.7–65.5)		1195	82.16(78.2–85.5)		7199	61.3(59.2–63.4)	

**Table 2:** Determinants of PNC use in Ethiopia (EDHS) 2000–2016 (n = 17,740).

Variables	Unadjusted OR	95%CI	*p* Value	Adjusted OR	95%CI	*p* Value
Predisposing Factors						
**Maternal education**						
No education	1.00					
Primary	2.57	2.16–3.05	*p* < 0.001	0.95	0.72–1.26	*p* = 0.75
Secondary	11.8	9.50–14.7	*p* < 0.001	1.19	0.80–1.77	*p* = 0.388
**Maternal Working status**						
Not working	1.00					
Professional	2.33	1.92–2.84	*p* < 0.001	1.02	0.77–1.35	*p* = 0.869
Agricultural	0.78	0.64–0.94	*p* = 0.013	1.13	0.87–1.48	*p* = 0.335
**Residence**						
Rural	1.00					
Urban	6.77	4.22–9.65	*p* < 0.001	2.99	1.43–3.45	*p* = 0.003
**Father’s education**						
No education	1.00					
Primary	1.53	1.28–1.83	*p* < 0.001	0.96	0.74–1.25	*p* = 0.781
Secondary	5.27	4.36–6.37	*p* < 0.001	0.86	0.62–1.20	*p* = 0.399
**Father’s working status**						
Not working	1.00					
Professional	1.57	1.07–2.31	*p* = 0.020	1.52	1.000094–2.34	*p* = 0.050
Agricultural	0.37	0.26–0.53	*p* < 0.001	1.32	0.904–1.943	*p* = 0.149
**Household wealth index**						
Poor	1.00					
Middle	1.92	1.58–2.33	*p* < 0.001	1.25	0.970–1.628	*p* = 0.083
Rich	7.71	6.34–9.38	*p* < 0.001	1.57	1.12–2.20	*p* = 0.008
**Mother’s age**						
15–24	1.00					
25–34	1.07	0.91–1.26	*p* = 0.349	1.042	0.784–1.385	*p* = 0.774
35–49	0.80	0.65– 0.99)	*p* = 0.046	0.832	0.573–1.220	*p* = 0.338
**Need factors**						
**ANC visit**						
None	1.00					
1–3	4.22	3.40–5.25	*p* < 0.001	1.911	1.36–2.66	*p* < 0.001
Four or more	12.1	9.92–14.9	*p* < 0.001	1.10	2.15–4.21	*p* < 0.001
**Place of delivery**						
Home	1.00					
Health facility	22.8	18.8–27.6	*p* < 0.001	2.56	1.048–6.25	*p* = 0.039
**Type of delivery assist**						
Health professional	1.00					
TBA	0.06	0.05–0.08	*p* < 0.001	0.218	0.085–0.559	*p* = 0.002
Other non-health professional	0.02	0.02– 0.03	*p* < 0.001	0.149	0.058–0.383	*p* < 0.001
**Birth interval**						
No previous birth	1.00					
<24months	0.34	0.26–0.44	*p* < 0.001	0.75	0.477–1.194	*p* = 0.230
≥24 months	0.56	0.48–0.66	*p* < 0.001	1.28	0.919–1.795	*p* = 0.141
**Enabling factors**						
**Accompany for medical help**						
Big problem	1.00					
Not a big problem	2.29	1.93–2.72	*p* < 0.001	0.99	0.786–1.268	*p* = 0.991
**Distance from the health facility**						
Big problem	1.00					
Not a big problem	3.68	3.14–4.32	*p* < 0.001	1.29	1.02–1.61	*p* = 0.028
**Permission for medical help**						
Big problem	1.00					
Not a big problem	1.79	1.48–2.17	*p* < 0.001	0.92	0.705–1.208	*p* = 0.563

**Table 3:** Determinants of PNC use in urban and rural Ethiopia (EDHS) 2000–2016 (n = 17,740).

	Urban Residence						Rural Residence					
Variables	Unadjusted OR	95%CI	*p* Value	Adjusted OR	95%CI	*p* Value	Unadjusted OR	95%CI	*p* value	Adjusted OR	95%CI	*p* Value
**Predisposing factor**												
**Maternal education**												
No education	1.00			1.00			1.00			1.00		
Primary	1.80	1.20–2.69	*p =* 0.004	0.63	0.35–1.13	*p* = 0.125	2.16	1.7–2.6	*p* < 0.001	1.06	0.77–1.4	*p =* 0.72
Secondary	4.00	2.73–5.85	*p* < 0.001	0.89	0.50–1.59	*p* = 0.711	6.47	4.3–9.5	*p* < 0.001	1.29	0.6–2.4	*p =* 0.44
**Maternal Working status**												
Not working	1.00			1.00			1.00			1.00		
Professional	1.27	0.91–1.79	*p =* 0.156	1.08	0.69–1.70	*p* = 0.714	1.73	1.32–2.26	*p* < 0.001	0.97	0.69–1.37	*p =* 0.90
Agricultural	0.74	0.41–1.31	*p* = 0.305	1.16	0.57–2.35	*p* = 0.680	0.93	0.75–1.15	*p =* 0.524	1.12	0.84–1.49	*p =* 0.423
**Father’s education**												
No education	1.00			1.00			1.00			1.00		
Primary	1.64	1.07–2.51	*p =* 0.023	1.17	0.63–2.16	*p* = 0.610	1.29	1.05–1.57	*p =* 0.012	0.92	0.68–1.23	*p =* 0.581
Secondary	2.92	2.07–4.11	*p* < 0.001	1.37	0.76–2.46	*p* = 0.291	2.45	1.83–3.28	*p* < 0.001	0.72	0.47–1.10	*p =* 0.132
**Father’s working status**												
Not working	1.00			1.00			1.00			1.00		
Professional	0.56	0.29 1.08	*p* =0.085	1.00	0.46–2.15	*p =* 0.998	1.43	0.90–2.26	*p* = 0.12	1.95	1.12–3.38	*p =* 0.017
Agricultural	0.47	0.24–0.90	*p* = 0.025	1.03	0.49–2.17	*p =* 0.934	0.512	0.33–0.77	*p =* 0.001	1.55	0.97–2.48	*p =* 0.066
**Marital status**												
Never married	1.00			1.00			1.00			1.00		
Formerly married	0.69	0.24–1.97	*p* = 0.493	1.05	0.50–2.19	*p =* 0.333	0.416	0.15–1.09	*p =* 0.075	1.40	0.73–2.70	*p =* 0.305
Currently married	1.08	0.45–2.59	*p* = 0.857				0.36	0.14–0.91	*p =* 0.032			
**Household wealth index**												
Poor	1.00			1.00			1.00			1.00		
Middle	2.51	1.00–6.29	*p* < 0.001	1.32	0.411–4.25	*p* = 0.083	1.75	1.43–2.14	*p* < 0.001	1.19	0.90–1.57	*p =* 0.201
Rich	4.25	1.83–9.84	*p* < 0.001	1.43	0.51–3.96	*p* = 0.008	3.62	2.76–4.76	*p* < 0.001	1.85	1.23–2.79	*p =* 0.003
**Mother’s age**												
15–24	1.00			1.00			1.00			1.00		
25–34	1.50	1.06–2.11	*p* = 0.020	1.17	0.70–1.95	*p* = 0.774	0.86	0.70–1.05	*p =* 0.150	1.03	0.71–1.49	*p =* 0.873
35–49	0.98	0.62–1.55	*p* = 0.961	0.57	0.25–1.29	*p* = 0.338	0.81	0.63–1.03	*p =* 0.096	0.96	0.62–1.49	*p =* 0.888
**Need factors**												
**ANC visit**												
None	1.00			1.00			1.00			1.00		
1–3	3.09	1.79–5.34	*p* < 0.001	0.77	0.39–1.50	*p* < 0.001	3.88	3.04–4.97	*p* < 0.001	2.27	1.55–3.32	*p* < 0.001
Four or more	7.73	4.71–12.6	*p* < 0.001	1.78	0.92–3.41	*p* < 0.001	8.58	6.66–11.0	*p* < 0.001	3.27	2.21–4.83	*p* < 0.001
**Place of delivery**												
Home	1.00			1.00			1.00			1.00		
Health facility	9.89	6.56–14.9	*p* < 0.001	2.11	0.63–7.01	*p* = 0.039	20.5	16.2–25.8	*p* < 0.001	2.68	0.81–8.86	*p =* 0.105
**Type of delivery assist**												
Health professional	1.00			1.00			1.00			1.00		
TBA	0.086	0.05–0.14	*p* < 0.001	0.185	0.053–0.648	*p* = 0.008	0.078	0.06–0.10	*p* < 0.001	0.22	0.06–0.76	*p =* 0.017
Other non-health professional	0.084	0.04–0.015	*p* < 0.001	0.181	0.046–0.706	*p =* 0.014	0.031	0.02–0.04	*p* < 0.001	0.14	0.04–0.51	*p =* 0.002
**Birth interval**												
No previous birth	1.00			1.00			1.00			1.00		
<24months	0.43	0.26–0.70	*p =* 0.001	0.953	0.45–2.01	*p =* 0.901	0.407	0.29–0.56	*p* < 0.001	0.67	0.37–1.20	*p =* 0.186
≥24 months	0.75	0.54–1.03	*p* = 0.079	1.78	1.08–2.95	*p =* 0.023	0.64	0.52–0.79	*p* < 0.001	1.11	0.71–1.72	*p =* 0.634
**Accompany for medical help**												
Big problem	1.00			1.00			1.00			1.00		
Not a big problem	1.27	0.92–1.76	*p =* 0.139	0.89	0.59–1.35	*p* = 0.605	1.87	1.51–2.32	*p* < 0.001	1.02	0.75–1.38	*p =* 0.883
**Distance from the health facility**												
Big problem	1.00			1.00			1.00			1.00		
Not a big problem	1.53	1.09–2.14	*p =* 0.014	1.18	0.75–1.88	*p* = 0.459	2.60	2.12–3.17	*p* < 0.001	1.35	1.03–1.76	*p =* 0.029
**Permission for medical help**												
Big problem	1.00			1.00			1.00			1.00		
Not a big problem	1.32	0.83–2.09	*p =* 0.234	0.66	0.343–1.27	*p =* 0.213	1.39	1.11–1.74	*p =* 0.004	0.97	0.72–1.31	*p =* 0.893

## Data Availability

The datasets used for this study are publicly available from the DHS program website (http://dhsprogram.com/data/).

## Abbreviations

ANC	Antenatal care
SDG	Sustainable Development Goal
MDG	Millennium Development Goal
WHO	World Health Organization
ICF	Inner City Fund
EDHS	Ethiopia Demographic and Health Survey
PNC	Postnatal care
CSA	Central Statistical Agency
MOH	Minster of Health
CIS	Confidence Interval
DHS	Demographic and Health Survey
SNNPR	Southern Nations, Nationalities, and People’s Region
BCC	Behavioural change communication
